# Structural maintenance of chromosome protein 1A exacerbates liver fibrosis by enhancing hepatic stellate cell activation and extracellular matrix synthesis via laminin subunit gamma 2 activation

**DOI:** 10.1002/ccs3.70067

**Published:** 2026-02-23

**Authors:** Dandan Wang, Ning Li, Ranyan Gao, Jiaxin Wang, Lingyi Xu, Fengchun Li, Xinyu Geng, Ram Prasad Chaulagain, Babalola Deborah Oluwaseun, Xiaoyu Zhang, Shuang Jin, Shizhu Jin

**Affiliations:** ^1^ Department of Gastroenterology and Hepatology The Second Affiliated Hospital of Harbin Medical University Harbin Heilongjiang China; ^2^ Department of Gastroenterology and Hepatology The First Hospital of Harbin Harbin Heilongjiang China; ^3^ Department of Infectious Diseases The Second Affiliated Hospital of Harbin Medical University Harbin Heilongjiang China; ^4^ Department of Gastroenterology and Hepatology The Third Affiliated Hospital of Qiqihar Medical University Qiqihar Heilongjiang China

**Keywords:** extracellular matrix accumulation, hepatic stellate cell, LAMC2, liver fibrosis, SMC1A

## Abstract

Liver fibrosis is characterized by an abnormal buildup of extracellular matrix (ECM), which is primarily produced by hepatic stellate cells (HSCs). Laminin subunit gamma 2 (LAMC2) is an ECM protein whose functional role in hepatic fibrosis remains to be fully elucidated. Herein, we examine how LAMC2 contributes to liver fibrosis and explore the molecular mechanisms in both animal and cellular models. LAMC2 was knocked down in C57BL/6J mice with CCl4‐induced liver fibrosis. Rescue experiments were conducted in sh‐LAMC2‐treated and recilisib (PI3K/Akt agonist)‐treated mice. The transcription factors associated with LAMC2 in liver fibrosis were predicted and verified. Transforming growth factor (TGF)‐β1‐stimulated LX‐2 cells (HSC line) were infected with lentiviral vectors for in vitro assays. LAMC2, which was enriched in the PI3K/Akt pathway, was increased in the liver tissues of mice treated with CCl4, and recilisib reversed LAMC2 knockdown‐mediated alleviation of liver fibrosis in these mice. LAMC2 transcription in activated HSCs was caused by structural maintenance of chromosome protein 1A (SMC1A). The inhibitory effect of SMC1A knockdown on ECM accumulation and HSC activation was mitigated by LAMC2 overexpression. This study provides new insights and highlights the promising potential of the SMC1A/LAMC2/PI3K/Akt axis as a therapeutic target for liver fibrosis.

## Graphical abstract

Structural maintenance of chromosome protein 1A activates the PI3K/Akt signaling pathway by promoting laminin subunit gamma 2 transcription, which facilitates hepatic stellate cell activation and extracellular matrix deposition, leading to liver fibrosis progression.

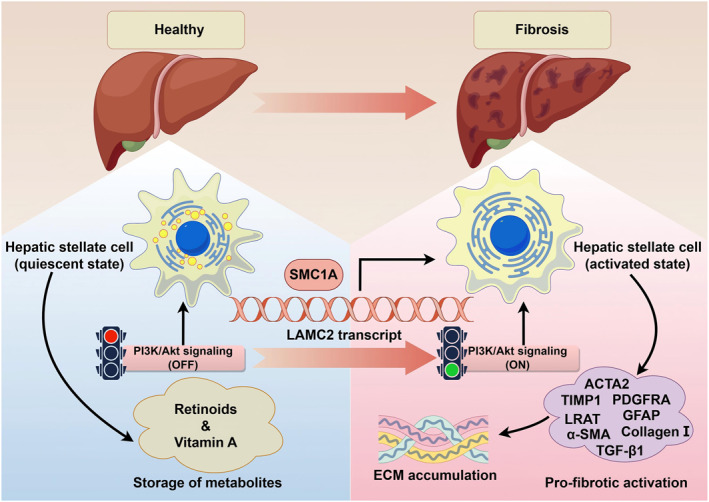

## 1 INTRODUCTION

Liver fibrosis involves a highly integrated series of molecular, cellular, and tissue processes that lead to excessive extracellular matrix (ECM) components. The related phenotypes are maintained by a population of myofibroblasts in the liver, and liver fibrosis is a critical pathological turning point at which chronic liver disease advances to cirrhosis and liver failure.^1^, ^2^ Accounting for approximately 15% of total intrinsic liver cells and 30% of the nonparenchymal cell population, hepatic stellate cells (HSCs) play a central role in fibrosis, acting as the key drivers of its progression.^3^ The physiological functions of HSCs include regulating retinoid storage and metabolism, secreting cytokines and growth factors, and transdifferentiating into profibrogenic myofibroblasts after liver injury.^4^ Therefore, understanding the mechanism underlying HSC activation and ECM accumulation is necessary for developing effective antifibrotic therapies.

The main ECM family members include various proteins and glycoproteins, including collagens, elastin, laminins, tenascins, proteoglycans, glycosaminoglycans, hyaluronan, and their corresponding cellular receptors.^5^ All laminins contain three distinct protein subunits (α, β, and γ), and 16 identified isoforms are formed through various combinations of five α chains (α1–α5), three β chains (β1–β3), and three γ chains (γ1–γ3), each exhibiting specific cellular and tissue expression.^6^ Laminin subunit gamma 2 (LAMC2), a component of laminin‐332 (also known as laminin‐5), has been identified as a biomarker for liver cancer.^7^ Moreover, it showed upregulation in the development of alcoholic hepatitis.^8^ In addition, elevated LAMC2 expression in vascular endothelial cells could play a role in the pathogenesis of fibrosis and chronic inflammation in keloids^9^; however, whether it regulates HSC activation, thereby controlling liver fibrosis, has not been reported. LAMC2 has been demonstrated to influence phosphatidylinositol 3‐kinase (PI3K)/protein kinase B (Akt) signaling pathway activity under different conditions,^10^, ^11^ and this metabolic pathway has been linked to the progression of chronic liver disease, liver fibrosis, liver cirrhosis, and hepatocellular carcinoma.^12^ In this study, by combining several bioinformatics tools, we determined that LAMC2, which is enriched in PI3K/Akt signaling, is differentially expressed in liver fibrosis and that structural maintenance of chromosome protein 1A (SMC1A) is the upstream transcription factor that manipulates its expression. SMCs belong to a large family of ring‐shaped complexes involved in numerous DNA processes; among SMCs, SMC1A plays roles in gene transcription regulation and genome organization.^13^ Therefore, we investigated the impact of the LAMC2‐mediated PI3K/Akt pathway on HSC activation, fibrotic behavior, and ECM accumulation and the molecular mechanisms of those processes.

## 2 MATERIALS AND METHODS

### 2.1 Animal study

The experimental procedures followed the Guide for the Care and Use of Laboratory Animals of the NIH. The animal ethics committee of the Second Affiliated Hospital of Harbin Medical University approved this study. Male C57BL/6J mice were fed in the Experimental Animal Center of the Second Affiliated Hospital of Harbin Medical University. All mice were adapted to the environment for 7 days with ad libitum access to food and water. They were assigned to various groups at random (*n* = 5/group except *n* = 10 for the sh‐NC group): normal, model, short hairpin RNA (sh)‐negative control (NC), sh‐LAMC2, dimethyl sulfoxide (DMSO) + sh‐LAMC2, recilisib + sh‐LAMC2, sh‐SMC1A, sh‐SMC1A + NC‐overexpression (oe), and sh‐SMC1A + LAMC2‐oe. To induce liver fibrosis, CCl4 (C119833, Aladdin) diluted 1:9 with corn oil was intraperitoneally injected into the mice at a dose of 1 mL/kg twice weekly for 6 weeks^14^; the same dosage of corn oil was intraperitoneally injected into mice in the normal group.

Lentiviral particles containing LAMC2, SMC1A, or control (sh‐NC) shRNA or LAMC2 overexpression or control (NC‐oe) plasmids were procured from VectorBuilder at a viral concentration of 1 × 10^9^ TU/mL. Two weeks after CCl4 treatment, mice in the appropriate groups were injected with the corresponding lentiviral particles via the tail vein (0.05 mL/mouse).^15^, ^16^ The PI3K/Akt agonist recilisib (10 mg/kg, HY‐101625, MedChemExpress) dissolved in DMSO was administered intraperitoneally once daily during the last 3 weeks of CCl4 treatment.^17^ Mice serving as controls received an equal volume of sterile DMSO via intraperitoneal injection according to the same schedule. Two days after the last injection, all mice were sacrificed by rapid cervical dislocation after intraperitoneal injection of 1.25% avertin (tribromoethanol) as an anesthetic, and peripheral blood and liver tissue were collected.

### 2.2 Cell culture and treatment

HSC LX‐2 cells (CL‐0560) were procured from Procell. They were grown in high‐glucose Dulbecco's modified Eagle medium (PM150210, Procell) containing 10% FBS and 1% penicillin‐streptomycin (all from Procell) and incubated at 37°C and 5% CO_2_. The cells were transduced with lentiviral particles containing shRNAs targeting SMC1A or LAMC2‐overexpression plasmids or with control lentivirus (1 × 10^9^ TU/mL, purchased from VectorBuilder) for 48 h and subsequently cultured in complete growth medium containing puromycin (2 μg/mL).^18^ LX‐2 cells were activated with 10 ng/mL transforming growth factor (TGF)‐β1 (100‐21; PeproTech Inc.) with PBS as a control for 24 h.^19^

### 2.3 IHC and histopathology

After 6 weeks of modeling, mouse liver tissues were fixed in formalin, embedded in paraffin, and sectioned (4‐μm thick) for standard immunohistochemistry (IHC) staining. Tissues were incubated with antibodies against LAMC2 (1:100, PK08311, Abmart Shanghai Co., Ltd.), α‐smooth muscle actin (α‐SMA, 1:500, GTX100034, GeneTex, Inc.), and collagen I (1:100, GTX20292, GeneTex) overnight at 4°C and with goat anti‐rabbit IgG (1:2500, ab97051, Abcam) at 37°C for 1 h. Then, immunoassays were performed using a DAB horseradish peroxidase color development kit (P0202, Beyotime Biotechnology Co., Ltd.). The chromogenic solution was adjusted to a ratio of 1:1, and the chromogenic reaction was terminated after the sections were washed twice with distilled water. Positive cells were quantified as a percentage of the total cell count in the analyzed samples.

The degree of liver fibrosis was assessed with a Masson's Trichrome Stain Kit (G1340, Solarbio). Tissue sections were stained with hematoxylin for 5 min, acid fuchsin for 10 min, and aniline blue for 1 min, followed by direct observation under a microscope.

The pathological changes in liver tissue were evaluated using an HE staining kit (C0105S, Beyotime). The section staining protocol involved 8 min of incubation in hematoxylin and 1 min of incubation in eosin.

### 2.4 RNA extraction and reverse transcription quantitative PCR (RT‐qPCR) analysis

Liver tissues were cut into small pieces (50–80 mg) and homogenized in 1 mL of TRIzol (R0016, Beyotime). After the culture medium was discarded, 1 mL of TRIzol was added to each 10 cm^2^ sample for total RNA extraction. Gene expression was analyzed by one‐step RT‐PCR using SuperScript IV UniPrime (12596100, Thermo Fisher Scientific). GAPDH was selected as the endogenous control. Relative mRNA expression was quantified via the 2^–ΔΔCt^ calculation approach. The sequences of primers can be found in Table 1.

**TABLE 1:** Primer sequences used in RT‐qPCR.

Gene	Species	Sequence
LAMC2	Mouse	FWD 5′‐CAGAGTTCAGGATACGAGCAGAC‐3′
		REV 5′‐GTCTGCCAATCTTGTAGCCTCC‐3′
ACTA2	Mouse	FWD 5′‐TGCTGACAGAGGCACCACTGAA‐3′
		REV 5′‐CAGTTGTACGTCCAGAGGCATAG‐3′
LAMA1	Mouse	FWD 5′‐TGTCTGCAAGCCAGGAGCTACA‐3′
		REV 5′‐GAGACAGAACGGCATCACCAAC‐3′
TIMP1	Mouse	FWD 5′‐TCTTGGTTCCCTGGCGTACTCT‐3′
		REV 5′‐GTGAGTGTCACTCTCCAGTTTGC‐3′
PDGFRA	Mouse	FWD 5′‐GCAGTTGCCTTACGACTCCAGA‐3′
		REV 5′‐GGTTTGAGCATCTTCACAGCCAC‐3′
GAPDH	Mouse	FWD 5′‐CATCACTGCCACCCAGAAGACTG‐3′
		REV 5′‐ATGCCAGTGAGCTTCCCGTTCAG‐3′
SMC1A	Human	FWD 5′‐CCTGAGACCTTCTTGCCTCTTG‐3′
		REV 5′‐GAGGTGGCTCATAGCGAATCAC‐3′
LAMC2	Human	FWD 5′‐TACAGAGCTGGAAGGCAGGATG‐3′
		REV 5′‐GTTCTCTTGGCTCCTCACCTTG‐3′
ACTA2	Human	FWD 5′‐CTATGCCTCTGGACGCACAACT‐3′
		REV 5′‐CAGATCCAGACGCATGATGGCA‐3′
LAMA1	Human	FWD 5′‐GAAGGTGACTGGCTCAGCAAGT‐3′
		REV 5′‐AGGCGTCACAACGGAAATCGTG‐3′
TIMP1	Human	FWD 5′‐GGAGAGTGTCTGCGGATACTTC‐3′
		REV 5′‐GCAGGTAGTGATGTGCAAGAGTC‐3′
PDGFRA	Human	FWD 5′‐GACTTTCGCCAAAGTGGAGGAG‐3′
		REV 5′‐AGCCACCGTGAGTTCAGAACGC‐3′
GAPDH	Human	FWD 5′‐GTCTCCTCTGACTTCAACAGCG‐3′
		REV 5′‐ACCACCCTGTTGCTGTAGCCAA‐3′

Abbreviations: ACTA2, actin alpha 2, smooth muscle; FWD, forward; LAMA1, laminin subunit alpha 1; LAMC2, laminin subunit gamma 2; PDGFRA, platelet‐derived growth factor receptor alpha; REV, reverse; RT‐qPCR, reverse transcription quantitative PCR; SMC1A, structural maintenance of chromosome protein 1A; TIMP1, TIMP metallopeptidase inhibitor 1.

### 2.5 Enzyme‐linked immunosorbent assay

Serum was prepared from the peripheral blood of mice using conventional methods. The serum concentrations of tumor necrosis factor (TNF)‐α (ab208348), interleukin (IL)‐1β (ab197742), and IL‐6 (ab222503) were measured using enzyme‐linked immunosorbent assay (ELISA) kits (all from Abcam).

### 2.6 Western blot analysis

LX‐2 cells and mouse hepatic tissues were lysed with cell lysis buffer (P0013, Beyotime), and protein levels were measured using an enhanced BCA protein assay kit (P0010S, Beyotime). After 10% SDS‐PAGE separation, the proteins were subjected to nitrocellulose membrane transfer. The membranes were incubated with primary antibodies against GAPDH (1:2500, ab9485, Abcam), LRAT (1:600, 12815‐1‐AP, Proteintech), GFAP (1:1000, PA5‐16291, Thermo Fisher Scientific), PI3K p85 alpha (1:1000, ab86714, Abcam), PI3K p85 alpha (phospho Y607) (1:500, ab182651, Abcam), Akt1 (1:1000, ab308107, Abcam), and Akt1 (phospho T308) (1:1000, ab308100, Abcam) diluted in blocking solution for 90 min at room temperature, followed by overnight incubation at 4°C. After rinsing, the membranes were incubated with an HRP‐conjugated secondary antibody (1:10,000, ab6721, Abcam) for 60 min at room temperature. After visualization using BeyoECL Plus (P0018S, Beyotime), signals were measured using ImageJ software.

### 2.7 Dual‐luciferase reporter assay

LX‐2 cells were seeded into 24‐well plates, and genomic segments containing the LAMC2 promoter region were amplified into the pGL4.20 basic vector (Promega Corporation). LX‐2 cells with sh‐NC and sh‐SMC1A were transfected with a reporter gene vector containing the LAMC2 promoter and a *Renilla* luciferase vector for 24 h. Following the manufacturer's instructions, the dual‐luciferase assay system (E2920, Promega) was employed to evaluate the collected cell samples, and *Renilla* luciferase activity was the control for normalization.

### 2.8 ChIP‐qPCR assay

Chromatin immunoprecipitation (ChIP) analysis was conducted using the ChIP assay kit (P2078, Beyotime) in accordance with the standard protocol to quantify the transcriptional activity of LAMC2. Fixed cells were harvested, lysed, and sonicated before immunoprecipitation with an anti‐SMC1A antibody (ab140493, Abcam) or IgG (ab37373, Abcam). To determine LAMC2 promoter enrichment, the precipitated DNA was amplified using PCR.

### 2.9 Immunofluorescence

Cells were fixed, permeabilized, and incubated with α‐SMA (1:500, GTX100034, GeneTex), collagen I (1:100, A22349, ABclonal), and SMC1A (1:500, ab243875, Abcam) for 12 h at 4°C, and with an immunofluorescence staining kit with FITC‐labeled goat anti‐rabbit IgG (P0186, Beyotime) for 1 h at room temperature. After that, the cell nuclei were counterstained with an antifade mounting medium containing DAPI (P0131, Beyotime). A laser fluorescence microscope (Olympus Optical Co., Ltd.) was used to photograph the cells, and the fluorescence intensity of the staining was measured using ImageJ software.

Paraffin‐embedded sections of mouse liver tissues were incubated with mouse anti‐α‐SMA (1:800, 67735‐1‐Ig, Proteintech) and rabbit anti‐SMC1A (1:500, ab243875, Abcam) or rabbit anti‐LAMC2 (1:100, PK08311, Abmart) at 4°C for 12 h, respectively, followed by incubation with FITC‐labeled goat anti‐rabbit IgG (H + L) and Cy3‐labeled goat anti‐mouse IgG (H + L) antibodies (A0521, Beyotime) at room temperature in the dark for 1 h. After mounting with DAPI‐containing mounting medium, images were captured under a fluorescence microscope. The fluorescence intensity of SMC1A or LAMC2 in activated HSCs (α‐SMA^+^) was assessed using ImageJ software.

### 2.10 Statistics

All data are presented as means ± standard deviations. An unpaired *t*‐test was employed for comparisons between two groups, whereas a one‐way analysis of variance was applied to compare more than two groups. Statistical significance was defined as *p* < 0.05. In terms of experimental reproducibility, the analyses incorporated data from at least three independent experiments. Prism 10.4.2 was used for all computations.

## 3 RESULTS

### 3.1 LAMC2 is strongly overexpressed in the liver tissue of CCl4‐treated mice

**FIGURE 1:**
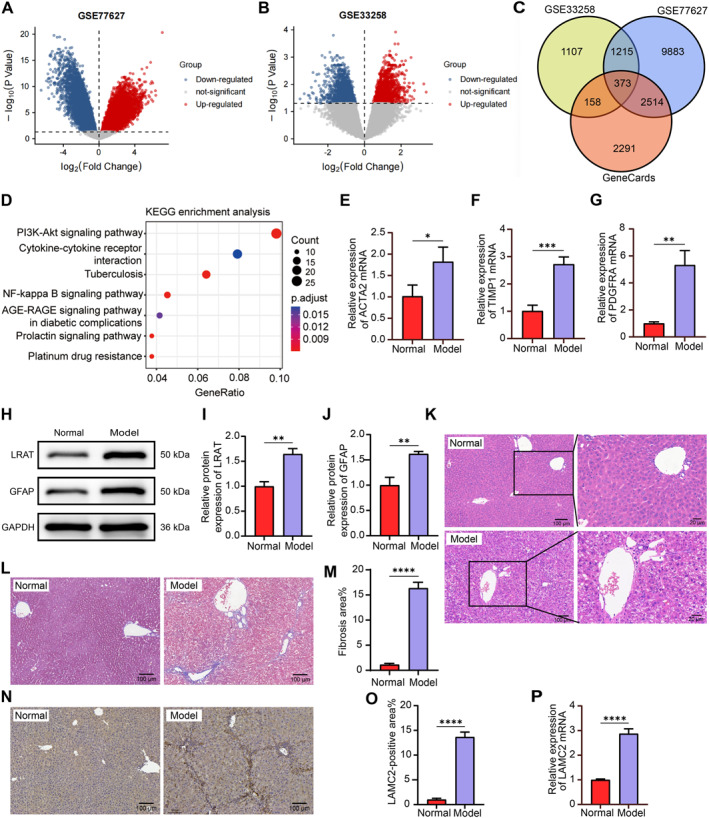
LAMC2 is significantly overexpressed in the liver tissues of cirrhotic mice. (A) Heatmap of differentially expressed genes in the GSE77627 dataset. (B) Heatmap of differentially expressed genes in the GSE33258 dataset. (C) The intersection of differentially expressed genes in the two GEO datasets and hub genes related to liver cirrhosis in the GeneCards database. (D) KEGG enrichment analysis of 373 intersecting genes. The expression of the profibrotic genes ACTA2 (E), TIMP1 (F), and PDGFRA (G) in the liver tissues of mice treated with DMSO (normal) or CCl4 (model), as examined by RT‐qPCR (*n* = 3). (H–J) Western blot analysis and relative protein expression levels of LRAT and GFAP in the liver tissues of mice (*n* = 3). (K) Analysis of fibrosis in the mouse liver lobule by HE staining (*n* = 5). (L, M) Analysis of fibrosis in the mouse liver lobule by Masson's trichrome staining (*n* = 5). (N, O) The LAMC2‐positive area in mouse liver samples was assessed by IHC (*n* = 5). (P) LAMC2 mRNA expression in mouse liver samples was assessed by RT‐qPCR (*n* = 3). Graphical data are presented as means ± SDs. *p < 0.05, **p < 0.01, ***p < 0.001, and ****p < 0.0001. An unpaired *t*‐test was used for statistical analysis. DMSO, dimethyl sulfoxide; IHC, immunohistochemistry; LAMC2, laminin subunit gamma 2; PDGFRA, platelet‐derived growth factor receptor alpha; RT‐qPCR, reverse transcription quantitative PCR; TIMP1, TIMP metallopeptidase inhibitor 1.

To elucidate the molecular processes underlying liver fibrosis, using the GSE77627 dataset from the GEO database, we examined the transcriptome profile of liver tissue from 14 histologically normal livers and 22 livers from patients with cirrhosis (Figure 1A, Supporting Information S1: Supplemental Material 1); using the GSE33258 dataset, we examined the gene expression profiles of stages F1 and F4 fibrotic livers (Figure 1B, Supporting Information S1: Supplemental Material 1). Genes associated with liver cirrhosis were subsequently downloaded from GeneCards (https://www.genecards.org/) (Supporting Information S1: Supplemental Material 1). jvenn (https://jvenn.toulouse.inrae.fr/app/example.html) was used to intersect the differentially expressed genes (adj. *p* < 0.05) from the GSE77627 and GSE33258 datasets, as well as the genes associated with cirrhosis. A total of 373 intersecting genes were found (Figure 1C, Supporting Information S1: Supplemental Material 2). The 373 intersecting genes were then evaluated for Kyoto Encyclopedia of Genes and Genomes (KEGG) enrichment using Hiplot (https://hiplot.com.cn/), which showed that these intersecting genes were mainly enriched in the PI3K/Akt signaling pathway (Figure 1D). We also examined 26 genes that were enriched in the PI3K/Akt signaling pathway. MAGI1 and LAMC2 have not been studied in the context of liver fibrosis. Both MAGI1 and LAMC2 are key genes among the intersecting genes (Supporting Information S1: Supplemental Material 2). The difference in LAMC2 expression (adj. *p* = 0.023444 [GSE33258], adj. *p* = 4.96E‐07 [GSE77627]) was more significant than that in MAGI1 expression (adj. *p* = 0.025206 [GSE33258], adj. *p* = 3.95E‐04 [GSE77627]); therefore, LAMC2 was chosen for subsequent analyses.

Profibrotic genes (ACTA2, TIMP1, and PDGFRA) were shown to be expressed in liver fibrosis model mice by RT‐qPCR. ACTA2 (Figure 1E), TIMP1 (Figure 1F), and PDGFRA (Figure 1G) were all overexpressed in the CCl4‐treated group relative to the normal group. Western blot results revealed greater levels of HSC activation markers (LRAT and GFAP) in the model mice than in the normal mice (Figure 1H–J). Moreover, HE staining (Figure 1K) and Masson's trichrome staining (Figure 1L,M) revealed marked fibrosis in the hepatic lobules of mice in the model group, further verifying the successful generation of the mouse model.

LAMC2 expression in mouse liver tissues was examined using IHC (Figure 1N,O) and RT‐qPCR (Figure 1P). As expected, the liver tissues of CCl4‐treated mice presented a much greater positive area and a greater level of LAMC2 mRNA expression than those of normal mice.

### 3.2 LAMC2 loss inhibits liver fibrosis and suppresses HSC activation

**FIGURE 2:**
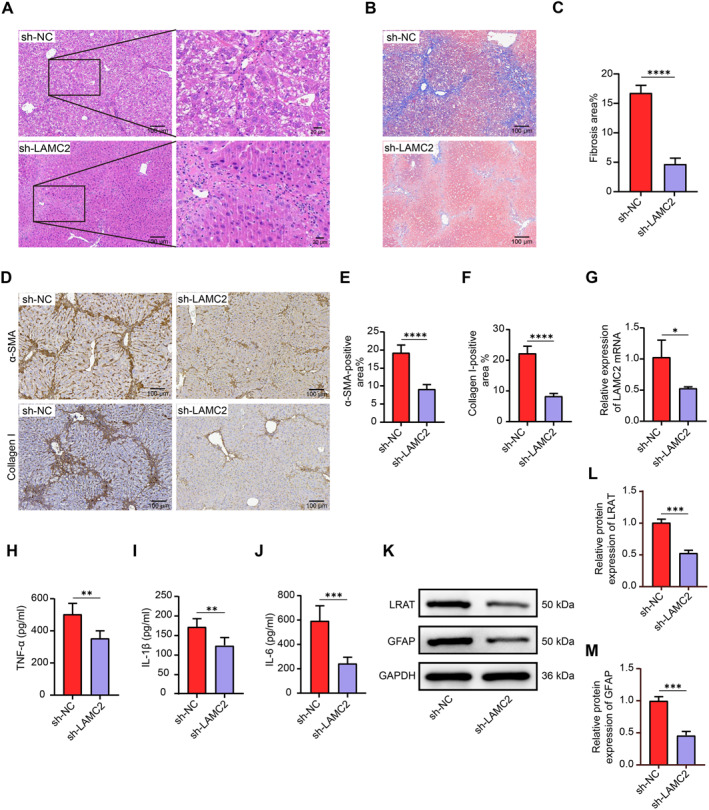
Poor expression of LAMC2 inhibits liver fibrosis and suppresses HSC activation in vivo. (A) Analysis of fibrosis in mouse liver tissues by HE staining (*n* = 5). (B, C) Analysis of fibrosis in mouse liver tissues by Masson's trichrome staining (*n* = 5). (D–F) The collagen I‐ and α‐SMA‐positive areas in mouse liver tissues were identified by IHC staining (*n* = 5). (G) The mRNA expression of LAMC2 in the liver tissues of mice injected with sh‐NC or sh‐LAMC2 was examined via RT‐qPCR (*n* = 3). The levels of the inflammatory factors TNF‐α (H), IL‐1β (I), and IL‐6 (J) in serum were measured using ELISAs (*n* = 5). (K‒M) The protein expression of LRAT and GFAP, which are markers of HSC activation, in mouse liver tissues was analyzed by Western blot (*n* = 3). Graphical data are presented as means ± SDs. *p < 0.05, **p < 0.01, ***p < 0.001, and ****p < 0.0001. An unpaired *t*‐test was used for statistical analysis. ELISAs, enzyme‐linked immunosorbent assays; HSC, hepatic stellate cell; IHC, immunohistochemistry; LAMC2, laminin subunit gamma 2; RT‐qPCR, reverse transcription quantitative PCR.

Liver fibrosis model mice were treated with sh‐NC or sh‐LAMC2. The in vivo impact of sh‐LAMC2 on the hepatic fibrosis phenotype was assessed by HE staining (Figure 2A) and Masson's trichrome staining (Figure 2B,C). sh‐LAMC2‐treated mice had less hepatic fibrosis. Changes in ECM accumulation were assessed by IHC staining. Collagen I‐ and α‐SMA‐positive areas were significantly reduced in liver tissues of mice after sh‐LAMC2 treatment (Figure 2D–F). RT‐qPCR results demonstrated that, compared with sh‐NC‐treated mice, sh‐LAMC2‐treated mice presented lower LAMC2 mRNA expression in liver tissue (Figure 2G). Serum levels of inflammatory cytokines, such as TNF‐α, IL‐1β, and IL‐6, were quantified using ELISAs. sh‐LAMC2‐treated mice had considerably decreased blood concentrations of TNF‐α (Figure 2H), IL‐1β (Figure 2I), and IL‐6 (Figure 2J). After LAMC2 knockdown, the expression of HSC activation markers in mouse liver tissues was significantly reduced, as determined by Western blot analysis (Figure 2K–M).

### 3.3 PI3K/Akt signaling pathway activation reverses the antifibrotic effects induced by sh‐LAMC2 in mice

**FIGURE 3:**
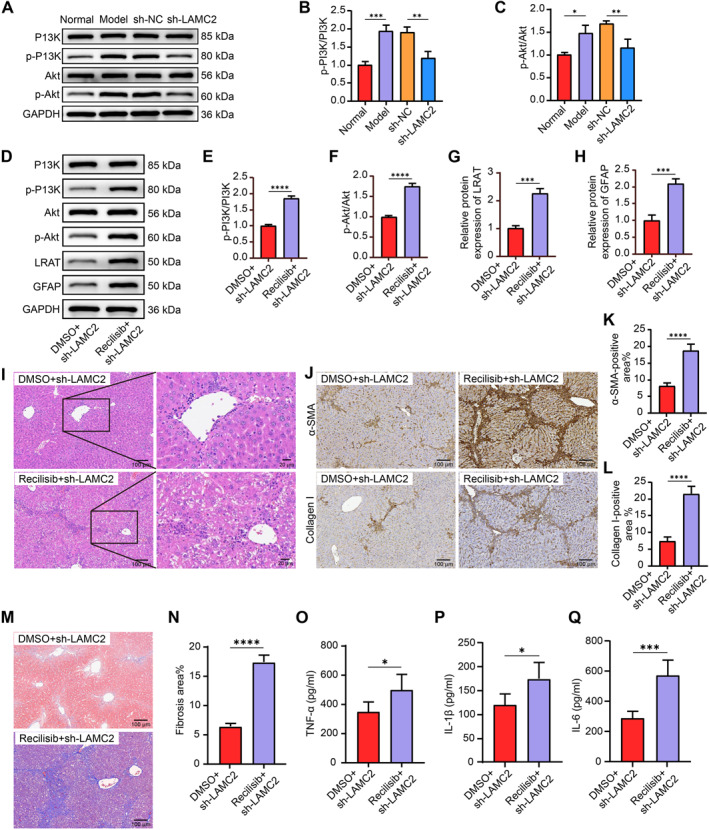
PI3K/Akt signaling activation exacerbates liver fibrosis in mice with sh‐LAMC2. (A–C) The total protein content and phosphorylation of PI3K and Akt in the liver tissues of normal and model mice and mice treated with sh‐NC or sh‐LAMC2 were examined using Western blot assays (*n* = 3). (D–H) The extent of PI3K and Akt phosphorylation and the protein expression of LRAT and GFAP in the liver tissues of mice treated with sh‐LAMC2 + DMSO or sh‐LAMC2 + recilisib were examined using Western blot assays (*n* = 3). (I) Analysis of fibrosis in mouse liver tissues via HE staining (*n* = 5). (J–L) The collagen I‐ and α‐SMA‐positive areas in mouse liver tissues were identified by IHC staining (*n* = 5). (M, N) Fibrosis in mouse liver tissues was analyzed via Masson's trichrome staining (*n* = 5). The levels of the inflammatory factors TNF‐α (O), IL‐1β (P), and IL‐6 (Q) in serum were measured using ELISAs (*n* = 5). Graphical data are presented as means ± SDs. *p < 0.05, **p < 0.01, ***p < 0.001, and ****p < 0.0001. An unpaired *t*‐test or one‐way ANOVA was used for statistical analysis. ANOVA, analysis of variance; DMSO, dimethyl sulfoxide; ELISAs, enzyme‐linked immunosorbent assays; IHC, immunohistochemistry.

Given that LAMC2 was enriched in PI3K/Akt signaling, we anticipated that pathway disruption would be linked to the antifibrotic actions of sh‐LAMC2. Western blot analysis revealed that liver tissues from CCl4‐treated mice presented considerably increased levels of PI3K and phosphorylated Akt, but that LAMC2 knockdown somewhat decreased these levels (Figure 3A–C).

We performed rescue experiments in a liver fibrosis model in mice using sh‐LAMC2 in combination with the PI3K/Akt agonist recilisib or the control agent DMSO. The blockade of PI3K/Akt signaling by sh‐LAMC2 was reactivated by recilisib (Figure 3D–F), which occurred concomitantly with increased expression of HSC activation markers (LRAT and GFAP) (Figure 3G,H). HE staining (Figure 3I) of liver tissues was performed. Mice that received recilisib had more severe liver fibrosis. IHC results revealed that, compared with DMSO treatment, recilisib treatment led to increased collagen I and α‐SMA levels in liver tissues (Figure 3J–L). Masson's trichrome staining (Figure 3M,N) also confirmed an increase in the area of fibrosis. Finally, ELISAs revealed that mice treated with recilisib presented considerably increased blood levels of inflammatory factors (Figure 3O–Q).

### 3.4 The transcription factor SMC1A promotes LAMC2 expression to promote HSC activation

**FIGURE 4:**
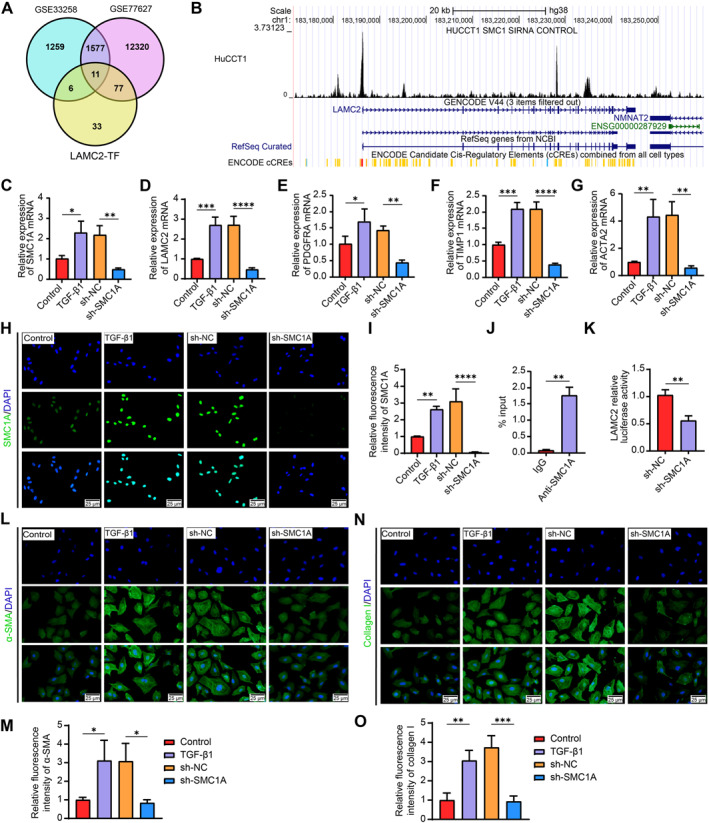
The transcription factor SMC1A promotes LAMC2 expression to induce HSC activation in vitro. (A) The transcription factors regulating LAMC2 in the hTFtarget database were intersected with differentially expressed genes in the two GEO datasets. (B) The binding peaks of SMC1A in the promoter region of LAMC2 were analyzed using the ChIP‐seq database. (C, D) The mRNA expression of SMC1A and LAMC2 in TGF‐β1‐stimulated LX‐2 cells and those preinfected with sh‐NC or sh‐SMC1A was examined via RT‐qPCR (*n* = 3). The expression of the profibrotic genes PDGFRA (E), TIMP1 (F), and ACTA2 (G) in LX‐2 cells was examined via RT‐qPCR (*n* = 3). The localization (H) and expression (I) of SMC1A in LX‐2 cells were examined via immunofluorescence experiments (*n* = 3). (J) The enrichment of SMC1A in the LAMC2 promoter region in LX‐2 cells was examined via ChIP‐PCR; immunoprecipitation was performed using an anti‐SMC1A antibody (*n* = 3). (K) Dual‐luciferase assay of the promoter transcriptional activity of LAMC2 in LX‐2 cells in the presence of sh‐SMC1A (*n* = 3). Representative immunofluorescence images and quantification of collagen I (L, M) and α‐SMA (N, O) in LX‐2 cells (*n* = 3). Graphical data are presented as means ± SDs. *p < 0.05, **p < 0.01, ***p < 0.001, and ****p < 0.0001. An unpaired *t*‐test or one‐way ANOVA was used for statistical analysis. ACTA2, actin alpha 2, smooth muscle; ANOVA, analysis of variance; ChIP, chromatin immunoprecipitation; HSC, hepatic stellate cell; LAMC2, laminin subunit gamma 2; PDGFRA, platelet‐derived growth factor receptor alpha; RT‐qPCR, reverse transcription quantitative PCR; SMC1A, structural maintenance of chromosome protein 1A; TIMP1, TIMP metallopeptidase inhibitor 1.

We downloaded transcription factors that target LAMC2 from hTFtarget (http://bioinfo.life.hust.edu.cn/hTFtarget/#!). We compared them to differentially expressed genes (adj. *p* < 0.05) in the GSE77627 and GSE33258 datasets using jvenn to gain insight into the upstream molecular processes of LAMC2. A total of 11 intersecting genes were found (Figure 4A): GRHL2, STAT1, NR3C1, BRD4, SMC1A, PBX1, ATF3, FOSL2, BCL11A, NIPBL, and NR2F2. GRHL2,^20^ STAT1,^21^ NR3C1,^22^ BRD4,^23^ PBX1,^24^ ATF3,^25^ and NR2F2^26^ have been implicated in liver fibrosis or HSC pathophysiology. Only four of the above genes have not been studied in liver cirrhosis. Among the four novel transcription factors, only SMC1A was significantly highly expressed in the GSE33258 (logFC = 0.801) and GSE77627 (logFC = 1.576659) datasets. In the UCSC ChIP‐seq data repository (https://genome.ucsc.edu/cgi‐bin/hgGateway), SMC1A displayed pronounced binding to the LAMC2 promoter sequence (Figure 4B).

LX‐2 HSCs were infected with sh‐NC or sh‐SMC1A and then treated with TGF‐β1. RT‐qPCR revealed that activated LX‐2 cells presented increased SMC1A and LAMC2 expression levels. Moreover, preinfection with sh‐SMC1A led to reduced SMC1A (Figure 4C) and LAMC2 mRNA expression (Figure 4D). Additionally, the mRNA expression of the profibrotic genes PDGFRA (Figure 4E), TIMP1 (Figure 4F), and ACTA2 (Figure 4G) significantly decreased after SMC1A knockdown. Immunofluorescence staining revealed SMC1A expression in the nuclei of HSCs. TGF‐β1 treatment enhanced SMC1A expression, whereas sh‐SMC1A significantly blocked SMC1A expression (Figure 4H,I). SMC1A was considerably concentrated in the promoter region of LAMC2 in LX‐2 cells, as determined by ChIP assays (Figure 4J). Additionally, in LX‐2 cells, sh‐SMC1A markedly reduced the transcriptional activity of the LAMC2 promoter (Figure 4K). α‐SMA (Figure 4L,M) and collagen I (Figure 4N,O) expression was substantially elevated in TGF‐β1‐treated LX‐2 cells, whereas SMC1A knockdown markedly decreased the expression of both of these proteins.

### 3.5 LAMC2 reverses the inhibitory effects of SMC1A knockdown on HSC activation and ECM deposition

**FIGURE 5:**
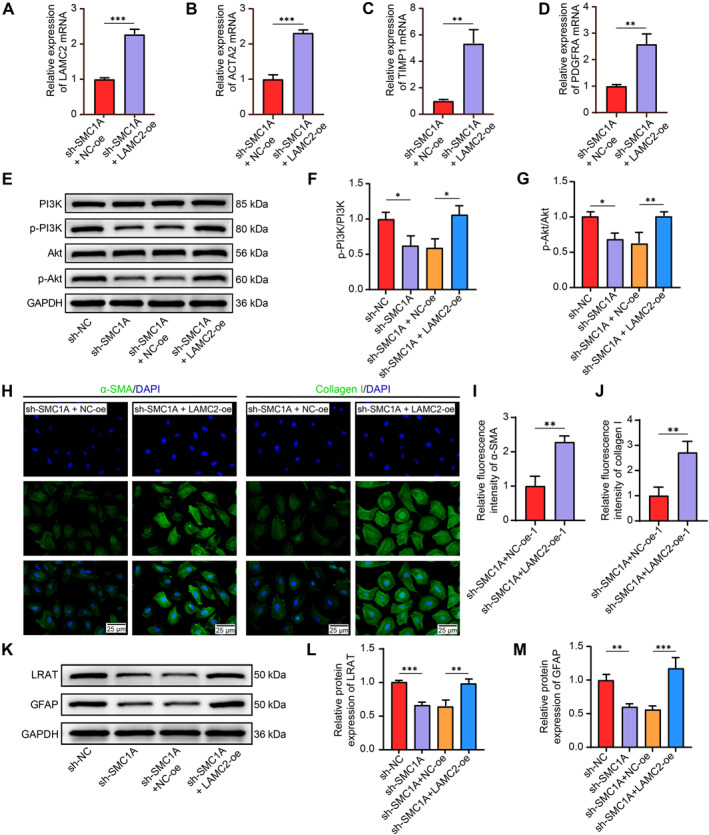
LAMC2 overexpression supports the profibrotic phenotype of HSCs in the presence of sh‐SMC1A. (A) The mRNA expression of LAMC2 in LX‐2 cells infected with sh‐SMC1A + NC‐oe or sh‐SMC1A + LAMC2‐oe was examined via RT‐qPCR (*n* = 3). The expression of the profibrotic genes ACTA2 (B), TIMP1 (C), and PDGFRA (D) in LX‐2 cells was examined via RT‐qPCR (*n* = 3). (E–G) The total protein content and phosphorylation of PI3K and Akt in LX‐2 cells were examined using Western blot assays (*n* = 3). (H–J) Representative immunofluorescence images (H) and quantification of collagen I (I) and α‐SMA (J) in LX‐2 cells (*n* = 3). (K‒M) The protein expression of LRAT and GFAP, which are markers of HSC activation, in LX‐2 cells was analyzed by Western blot assays (*n* = 3). Graphical data are presented as means ± SDs. *p < 0.05, **p < 0.01, and ***p < 0.001. An unpaired *t*‐test or one‐way ANOVA was used for statistical analysis. ACTA2, actin alpha 2, smooth muscle; ANOVA, analysis of variance; HSCs, hepatic stellate cells; LAMC2, laminin subunit gamma 2; PDGFRA, platelet‐derived growth factor receptor alpha; RT‐qPCR, reverse transcription quantitative PCR; SMC1A, structural maintenance of chromosome protein 1A; TIMP1, TIMP metallopeptidase inhibitor 1.

To confirm that LAMC2 is a downstream target of SMC1A, we treated LX‐2 cells with sh‐SMC1A in combination with LAMC2‐oe or NC‐oe. RT‐qPCR revealed that LAMC2‐oe significantly increased LAMC2 mRNA expression in LX‐2 cells (Figure 5A). The mRNA expression of the profibrotic genes ACTA2 (Figure 5B), TIMP1 (Figure 5C), and PDGFRA (Figure 5D) significantly increased after ectopic LAMC2 expression. Consistently, PI3K/Akt signaling blocked by sh‐SMC1A was reactivated by LAMC2 overexpression (Figure 5E–G). Upregulated LAMC2 expression in the presence of sh‐SMC1A increased the staining intensities of α‐SMA and collagen I (Figure 5H–J), together with the protein levels of LRAT and GFAP (Figure 5K–M), in LX‐2 cells.

### 3.6 LAMC2 overexpression aggravates liver fibrosis in mice treated with sh‐SMC1A

**FIGURE 6:**
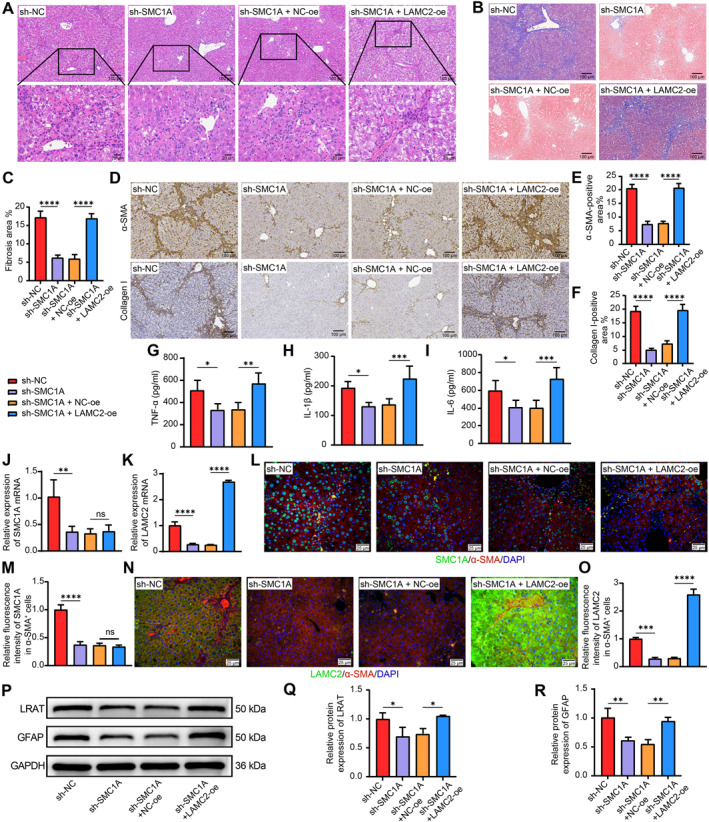
LAMC2 overexpression accentuates liver fibrosis in mice treated with sh‐SMC1A. (A) HE analysis of fibrosis in liver tissues from mice treated with sh‐SMC1A alone or sh‐SMC1A + NC‐oe/LAMC2‐oe (*n* = 5). (B, C) Analysis of fibrosis in mouse liver tissues via Masson's trichrome staining (*n* = 5). (D–F) The collagen I‐ and α‐SMA‐positive areas in mouse liver tissues were identified by IHC staining (*n* = 5). The levels of the inflammatory factors TNF‐α (G), IL‐1β (H), and IL‐6 (I) in serum were measured by ELISAs (*n* = 5). The mRNA expression of SMC1A (J) and LAMC2 (K) in liver tissues was examined via RT‐qPCR (*n* = 3). (L–O) The protein expression of SMC1A and LAMC2 in activated HSCs (α‐SMA^+^) was examined via immunofluorescence staining (*n* = 3). (P–R) The protein expression of LRAT and GFAP, which are markers of HSC activation, in mouse liver tissues was analyzed by Western blot (*n* = 3). Graphical data are presented as means ± SDs. *p < 0.05, **p < 0.01, ***p < 0.001, and ****p < 0.0001. A one‐way ANOVA was used for statistical analysis. ANOVA, analysis of variance; ELISAs, enzyme‐linked immunosorbent assays; HSC, hepatic stellate cell; IHC, immunohistochemistry; LAMC2, laminin subunit gamma 2; RT‐qPCR, reverse transcription quantitative PCR; SMC1A, structural maintenance of chromosome protein 1A.

CCl4‐treated mice received sh‐NC, sh‐SMC1A, sh‐SMC1A + NC‐oe, or sh‐SMC1A + LAMC2‐oe. Liver samples were stained with HE (Figure 6A) and Masson's trichrome (Figure 6B,C). The sh‐SMC1A group presented reduced hepatic fibrosis compared with the sh‐NC group. The sh‐SMC1A + LAMC2‐oe group exhibited significantly more severe hepatic fibrosis than the sh‐SMC1A + NC‐oe group. SMC1A knockdown reduced the number of regions positive for collagen I and α‐SMA, whereas LAMC2‐oe increased the number of such regions (Figure 6D–F). The serum inflammatory factor contents were subsequently analyzed by ELISAs. Levels of TNF‐α (Figure 6G), IL‐1β (Figure 6H), and IL‐6 (Figure 6I) were reduced in the sh‐SMC1A group relative to the sh‐NC group; after the administration of LAMC2‐oe, their levels noticeably increased. Mice injected with sh‐SMC1A alone presented substantially lower liver SMC1A and LAMC2 mRNA expression levels, according to RT‐qPCR (Figure 6J). After the administration of LAMC2‐oe, LAMC2 mRNA expression was markedly restored (Figure 6K). In activated HSCs (α‐SMA^+^), sh‐SMC1A‐mediated SMC1A suppression also resulted in reduced LAMC2 expression in HSCs, whereas LAMC2‐oe effectively rescued LAMC2 expression in HSCs (Figure 6L–O). Western blot results revealed that HSC activation was significantly hampered by sh‐SMC1A but was reactivated by LAMC2 overexpression (Figure 6P–R).

## 4 DISCUSSION

**FIGURE 7:**
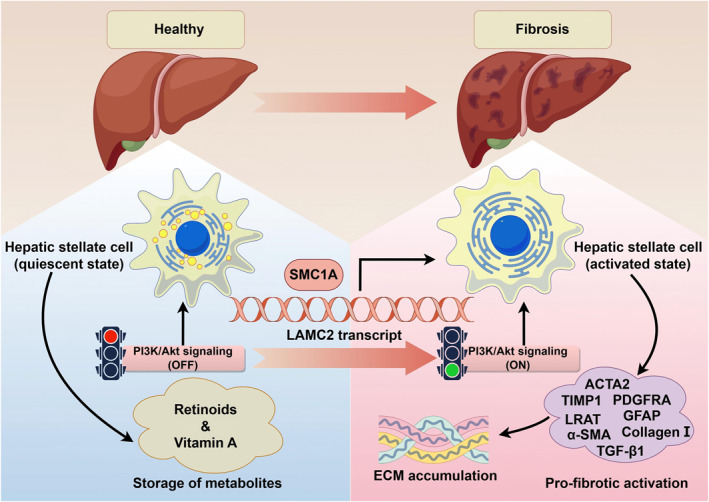
Overview diagram of the mechanism. SMC1A activates the PI3K/Akt signaling pathway by promoting LAMC2 transcription, which facilitates HSC activation and ECM deposition, leading to liver fibrosis progression. This figure was drawn using Figdraw (https://www.figdraw.com/#/). ECM, extracellular matrix; HSC, hepatic stellate cell; LAMC2, laminin subunit gamma 2; SMC1A, structural maintenance of chromosome protein 1A.

Worldwide, in more than 450 million individuals, fibrosis may progress to cirrhosis, which often leads to terminal liver disease, for which liver transplantation is currently the only curative option.^27^ Therefore, alternative treatments are necessary owing to limitations in organ transplantation. In this study, we provide evidence that SMC1A silencing ameliorates CCl4‐induced liver fibrosis by partially preventing HSC activation and ECM deposition by downregulating LAMC2 expression. Surprisingly, the antifibrotic impact of LAMC2 knockdown in vivo was hampered by PI3K/Akt signaling activation (Figure 7).

ACTA2 and PDGFRA are liver fibrosis‐specific genes.^28^, ^29^ The complex process of HSC activation facilitates their phenotypic shift from quiescence to a proliferative state, giving rise to excessive collagen and other fiber production, which ultimately accelerates the progression of liver fibrosis.^30^ HSCs are activated by many stimuli, including the profibrogenic cytokine TGF‐β1, and HSCs during activation express α‐SMA and ECM components, including collagen and fibronectin, and modulate ECM turnover by generating degrading enzymes (TIMPs).^31^ LRAT is dynamically distributed in HSC subtypes and is strongly expressed in some HSC subclusters,^32^ and GFAP has been shown to serve as a marker of HSCs in addition to its classic role as an astrocyte marker.^33^ The success of liver fibrosis induction was confirmed in this study by ACTA2, TIMP1, and PDGFRA mRNA overexpression and LRAT and GFAP protein expression in the liver tissues of CCl4‐treated mice. We confirmed that CCl4‐treated mice had increased liver LAMC2 expression, which somewhat supported our initial prediction.

TNF‐α, IL‐1, and IL‐6, among other cytokines, have a proinflammatory role and contribute to liver cell necrosis. This, in turn, leads to the development of fibrosis.^34^ Our in vivo observation that lentiviral particles containing sh‐LAMC2 decreased the levels of inflammatory cytokines (TNF‐α, IL‐1β, and IL‐6) and fibrosis markers (LRAT and GFAP) suggested that LAMC2 knockdown had antifibrotic effects. It has been revealed that forced expression of the LAMC2 monomer in hepatocytes adjacent to hepatic progenitor cells resulted in enhanced tumorigenicity, cell proliferation, and migration in immortalized hepatocytes.^35^ Recent research has demonstrated that LAMC2 is crucial for ECM deposition and ECM interaction with myofibroblasts, thereby promoting fibrosis in the heart.^36^ However, the function of LAMC2 in liver fibrosis has not been elucidated. The expression levels of total and phosphorylated forms of PI3K and Akt proteins in mouse liver tissues were assessed because LAMC2 is expected to be enriched in the PI3K/Akt signaling pathway. LAMC2 knockdown reduced PI3K/Akt signaling overactivation in the liver fibrosis model mice. Additionally, the PI3K/Akt agonist recilisib reversed the anti‐inflammatory and antifibrotic effects of sh‐LAMC2. Because PI3K/Akt plays a pivotal role in pulmonary fibrosis,^37^ the main findings of this study might offer a new understanding of how to manage fibroproliferative disorders.

Coskun et al. reported that the transcription factor CDX2 directly regulated LAMC2 expression through interactions with elements in the LAMC2 promoter region, thereby regulating active inflammation in colonic epithelial cells.^38^ SMC1A was identified as a transcription factor of LAMC2 due to its overexpression in liver fibrosis. SMC1A overexpression has been reported to rescue impaired tumor growth in liver cancer cells.^39^ Furthermore, during colorectal cancer liver metastasis, SMC1A overexpression attracted tumor‐associated fibroblasts, which then induced the secretion of the inflammatory factors TNF‐α and IL‐1β^40^; however, its regulatory role in HSCs and liver fibrosis has not been reported. It has been reported that p‐Akt expression is reduced after SMC1A knockdown in breast cancer cells,^41^ indicating the modulatory effects of SMC1A on PI3K/Akt signaling. Considering that the existing evidence regarding SMC1A is focused on cancers, revealing the effects of SMC1A on liver fibrosis through the LAMC2/PI3K/Akt axis is novel. Here, in vitro research revealed that LAMC2 overexpression reversed the inhibitory effects of SMC1A knockdown on the PI3K/Akt pathway, ECM deposition, and HSC activation. CCl4‐treated mice were used to replicate the in vitro results in vivo.

## 5 CONCLUSION

The results highlight the SMC1A/LAMC2/PI3K/Akt axis as a possible target for antifibrotic treatments. These interventions may inhibit fibrotic progression by targeting activated HSCs and ECM deposition, offering valuable treatment options for liver fibrosis. However, this study only proved that PI3K/Akt signaling is a downstream effector of LAMC2. Because knocking down LAMC2 decreased focal adhesion kinase (FAK) phosphorylation and downregulated ITGB4 in hepatocellular carcinoma cells^42^ and activated FAK can phosphorylate PI3K and Akt, thereby modulating the junction and hub of several intracellular pathways during HSC activation,^43^ we need further research to clarify the specific mechanism of LAMC2 in regulating PI3K/Akt signaling.

## AUTHOR CONTRIBUTIONS

**Dandan Wang**: Writing—original draft; validation; software; resources; methodology; investigation; formal analysis; data curation; conceptualization. **Ning Li**: Writing—review and editing; data curation; conceptualization. **Ranyan Gao**: Writing—review and editing; methodology; conceptualization. **Jiaxin Wang**: Validation; software; methodology; data curation. **Lingyi Xu**: Validation; software; methodology; data curation. **Fengchun Li**: Validation; software; data curation. **Xinyu Geng**: Writing—review and editing; conceptualization. **Ram Prasad Chaulagain**: Validation; software; data curation. **Babalola Deborah Oluwaseun**: Validation; software; data curation. **Xiaoyu Zhang**: Validation; software; data curation. **Shuang Jin**: Validation; software; methodology; data curation. **Shizhu Jin**: Writing—review and editing; funding acquisition; conceptualization.

## CONFLICT OF INTEREST STATEMENT

The authors declare no conflicts of interest.

## ETHICS STATEMENT

All animal experiments complied with the experimental guidelines established by the Experimental Animal Center of the Second Affiliated Hospital of Harbin Medical University (Ethics Project Number: YJSDW2024‐102).

## INFORMED CONSENT

N/A.

## REGISTRY AND THE REGISTRATION NO. OF THE STUDY/TRIAL:

N/A.

## HUMAN STUDIES:

N/A.

## Supporting information

Supporting Information S1

## Data Availability

The datasets used and/or analyzed in this study are available from the corresponding authors upon reasonable request.
