# Supplementary material for: Structural maintenance of chromosome protein 1A exacerbates liver fibrosis by enhancing hepatic stellate cell activation and extracellular matrix synthesis via laminin subunit gamma 2 activation
Source: J Cell Commun Signal. 2026 Feb 23;20(1):e70067. doi: 10.1002/ccs3.70067 (PMC12928013; doi:10.1002/ccs3.70067)
Supplement: Supplementary file 1 — Supporting Information S1 [file CCS3-20-e70067-s001.zip › revised supplementary file/Supplemental material 1/caption for supplemental material 1.docx]

DEG analyses of GEO datasets (GSE77627, GSE33258) and liver cirrhosis-related genes from GeneCards.
