# Supplementary material for: Structural maintenance of chromosome protein 1A exacerbates liver fibrosis by enhancing hepatic stellate cell activation and extracellular matrix synthesis via laminin subunit gamma 2 activation
Source: J Cell Commun Signal. 2026 Feb 23;20(1):e70067. doi: 10.1002/ccs3.70067 (PMC12928013; doi:10.1002/ccs3.70067)
Supplement: Supplementary file 1 — Supporting Information S1 [file CCS3-20-e70067-s001.zip › revised supplementary file/Supplemental material 2/caption for supplemental material 2.docx]

Jvenn intersection analysis of GSE77627/GSE33258 DEGs and liver cirrhosis-associated genes: 373 intersecting genes.
